# Early Epidemiological Features of COVID-19 in Nepal and Public Health Response

**DOI:** 10.3389/fmed.2020.00524

**Published:** 2020-08-11

**Authors:** Santosh Dhakal, Surendra Karki

**Affiliations:** ^1^W. Harry Feinstone Department of Molecular Microbiology and Immunology, Johns Hopkins Bloomberg School of Public Health, Baltimore, MD, United States; ^2^Department of Epidemiology and Public Health, Himalayan College of Agricultural Sciences and Technology, Kirtipur, Nepal

**Keywords:** severe acute respiratory syndrome coronavirus-2, coronavirus disease 2019, epidemiology, public health response, Nepal

## Abstract

Coronavirus disease 2019 (COVID-19), caused by Severe Acute Respiratory Syndrome Coronavirus 2 (SARS-CoV-2), was first reported in late 2019 from Wuhan, China. Considering COVID-19's alarming levels of spread and severity, the World Health Organization (WHO) declared a global pandemic on March 11, 2020. The first case of COVID-19 in Nepal was reported on January 23, 2020. The Government of Nepal implemented different public health measures to contain COVID-19, including border closures and a countrywide lockdown. We collected the daily data provided by the Ministry of Health and Population (MoHP) of the Government of Nepal and illustrated the early epidemiological characteristics of COVID-19 in Nepal. By May 31, 2020, 1,572 cases and eight deaths were reported in Nepal associated with COVID-19. The estimate of prevalence for COVID-19 among tested populations was 2.25% (95% CI: 2.15–2.37%) and case-fatality rate was 0.5%. The majority of the cases were young males (*n* = 1,454, 92%), with overall average age being 30.5 years (ranging from 2 months to 81 years) and were mostly asymptomatic. There were only five cases from three districts until the end of March, but cases surged from April and spread to 57 out of 77 districts of Nepal by the end of May 2020 despite the continuous lockdown. Most of these cases are from the southern plains of Nepal, bordering India. As the effect of COVID-19 is expected to persist longer, the Government of Nepal should make appropriate strategies for loosening lockdowns in a phase-wise manner while maintaining social distancing and personal hygiene and increasing its testing, tracking, and medical capacity.

## Introduction

Coronaviruses (CoVs) are enveloped, positive-sense, single-stranded RNA viruses with a comparatively larger genome size (30 Kb), belonging to the order *Nidovirales*, family *Coronaviridae*, and subfamily *Coronavirinae* (1). The subfamily is further divided into four genera: alpha, beta, gamma, and delta coronaviruses. Those infecting mammals fall within alpha and beta CoVs (2). When contracted by farm animals, CoVs are known to cause severe economic losses for a considerable time. Transmissible Gastroenteritis Virus (TGEV) and Porcine Epidemic Diarrhea Virus (PEDV) in pigs, and Bovine Coronaviruses (BCoVs) in cattle are a few such examples (3, 4). The PEDV outbreak in the US pig industry in 2013 was characterized by severe gastroenteritis in piglets. This outbreak killed over 7 million pigs within a year, which was 10% of the total pig population in the US (4). CoVs also cause Infectious Bronchitis in poultry, resulting in huge economic losses in the poultry industry. They are also transmissible to dogs and cats. In humans, CoVs (HCoV-NL63, HCoV-229E, HCoV-OC43, and KHU1) were traditionally known to cause mild respiratory infections until the emergence of Severe Acute Respiratory Syndrome Coronavirus (SARS-CoV) (5). SARS-CoV emerged from Guangdong Province, China, in November 2002 and spread rapidly to at least 27 countries, leading to over 8,000 reported cases and over 750 deaths, with about a 10% case-fatality rate (6). Within a decade of the SARS-CoV epidemic, another novel coronavirus infection was reported from Saudi Arabia, in June 2012 (6). This virus, later named Middle East Respiratory Syndrome Coronavirus (MERS-CoV), had around a 34% case-fatality rate and resulted in a total of nearly 2,500 laboratory-confirmed cases and over 850 associated deaths from 27 countries as of November 2019 (7). In December 2019, a series of viral pneumonia cases were reported from Wuhan, Hubei Province, China. The causative agent, a novel beta coronavirus, was first named as 2019 novel coronavirus (2019-nCoV) (8). 2019-nCoV was ultimately renamed as Severe Acute Respiratory Syndrome Coronavirus 2 (SARS-CoV-2) and the disease, characterized by symptoms including fever, shortness of breath, cough, fatigue, and pneumonia, was named Coronavirus Disease 2019 (COVID-19) by the World Health Organization (WHO) (9).

On 30th January 2020, the WHO declared that the COVID-19 outbreak constituted a Public Health Emergency of International Concern (PHEIC), which was eventually declared a global pandemic on 11th March 2020, owing to the alarming levels of spread and severity of COVID-19 (10). It is most likely that, similar to SARS and MERS coronaviruses, SARS-CoV-2 also originated from bat reservoirs. Studies have shown around an 80% genetic sequence homology between SARS-CoV-2 and SARS-CoV, while the resemblance of SARS-CoV-2 with bat coronaviruses is over 95% (11, 12). Bat coronaviruses require intermediate animal hosts before the spillover occurs in humans. For SARS-CoV and MERS-CoV, palm civet and dromedary camels, respectively, were found to serve as intermediate hosts (6). Pangolin coronaviruses had over a 90% similarity to SARS-CoV-2 but evidence contrasts regarding the possibility of pangolins being the intermediate host (13, 14). SARS-CoV-2 can infect animals including ferrets, domestic cats, tigers, and rhesus macaques either naturally or experimentally, but the actual intermediate host which contributed in virus transmission dynamics is not known yet (15–17).

As per the WHO's situation report from 31st May 2020, more than 5.9 million cases of COVID-19 were reported globally, with over 365,000 deaths (18). The US alone has reported more than 1.7 million cases and over 100,000 deaths (19). Other countries most severely affected with higher number of cases of COVID-19 include Brazil, Russia, Spain, the UK, India, Italy, Peru, Germany, and Turkey (9). Early epidemiological studies from China and the US indicated that older age and patients with underlying health conditions were at greater risk of hospitalization, intensive care unit (ICU) admission, and death due to COVID-19 (20, 21). A recent retrospective study from New York also showed that older age and chronic pulmonary and cardiac diseases were independently associated with in-hospital mortality with COVID-19 (22).

As of May 31, 2020, COVID-19 has been reported from 10 of 11 member countries in the WHO South-East Asia region. The highest number of cases have been reported from India, Bangladesh, and Indonesia (23). In Nepal, the first case of COVID-19 was officially reported on 23rd January 2020 in a 32-year-old man who returned from Wuhan, China (24). The second case was detected after two months on 23rd March. By May 31, 1,572 cases and eight deaths were reported from Nepal (25). The increasing situation of COVID-19 will be challenging for countries like Nepal where the health infrastructure is fragile and less equipped. In Nepal, there are only 194 hospitals with ICU facilities, with a capacity of 26,930 hospital beds, 3,076 isolation beds, 1,595 ICU beds, and 840 ventilators. In total, 111 hospitals run COVID-19 clinics while 13 hospitals are designated as level-I COVID-19 hospitals, 12 hospitals as level-II COVID-19 hospitals, and three hospitals as level-III COVID hospitals (26). In this article, we describe the early epidemiological features of COVID-19 in Nepal, its spatiotemporal distribution, the public health response taken by the Government of Nepal, and the way forward.

## Methods

### Study Design

This is a descriptive epidemiological study to highlight the early epidemiological features of COVID-19 cases in Nepal.

### Study Area

Nepal is a landlocked country surrounded by India in the south, east, and west and China in the north. Nepal has a population of around 30 million (27). Politically, Nepal is divided into seven provinces, 77 districts, and 753 local bodies. Geographically, it is divided into Terai (southern plains bordering to India), hills, and mountains (Himalayan range).

### Data Collection

The COVID-19 cases and Reverse Transcriptase Polymerase Chain Reaction (RT-PCR) testing data for this study were compiled using the publicly available official situation reports of the Ministry of Health and Population (MoHP) of the Government of Nepal (25). The MoHP made these data public through daily press meets and through national television broadcasts and MoHP's social media page. The daily situation reports are available from MoHP's website: https://drive.google.com/drive/folders/1QhLMbT76t6Zu1sFy5qlB5aoDbHVAcnHx. This study includes data from January 23, 2020 to May 31, 2020 to understand the early epidemiological features of COVID-19 cases in Nepal. COVID-19 cases in Nepal, as defined by the MoHP, included any individual who had RT-PCR tested positive for SARS-CoV-2 virus infection.

### Statistical Analysis

The daily data were collated in Microsoft Excel 2016. The graphs were created using the same version of Microsoft Excel. Descriptive statistics of the age distribution of confirmed COVID-19 cases such as the mean, median, minimum, maximum, standard deviation, and quartiles were calculated using the Epi Info version 7.2.3.1 developed by the Center for Disease Control and Prevention (CDC) of the United States (https://www.cdc.gov/epiinfo/index.html).

The choropleth maps, which helps to show the spatial patterns by shading the geographical areas in different colors, were created using the open-access software, QGIS version 3.10.3 (https://www.qgis.org/en/site/) to show the spatial distribution of COVID-19 cases in Nepal in three time periods (January-March; April and May 2020). We aggregated January-March as there were only a few cases (five) in total by the end of March.

## Results

**Figure 1:**
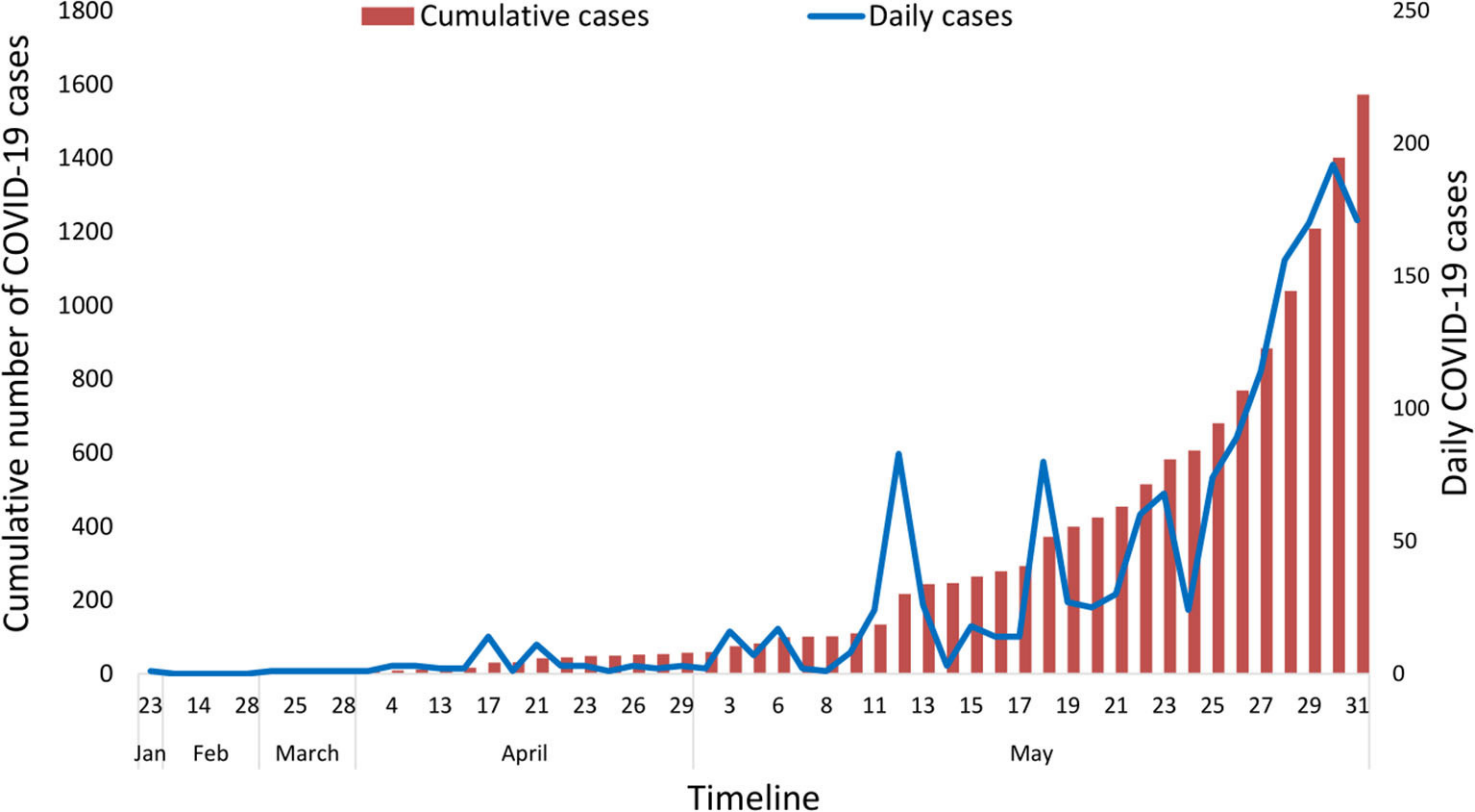
Epidemic curve of COVID-19 cases in Nepal.

**Figure 2:**
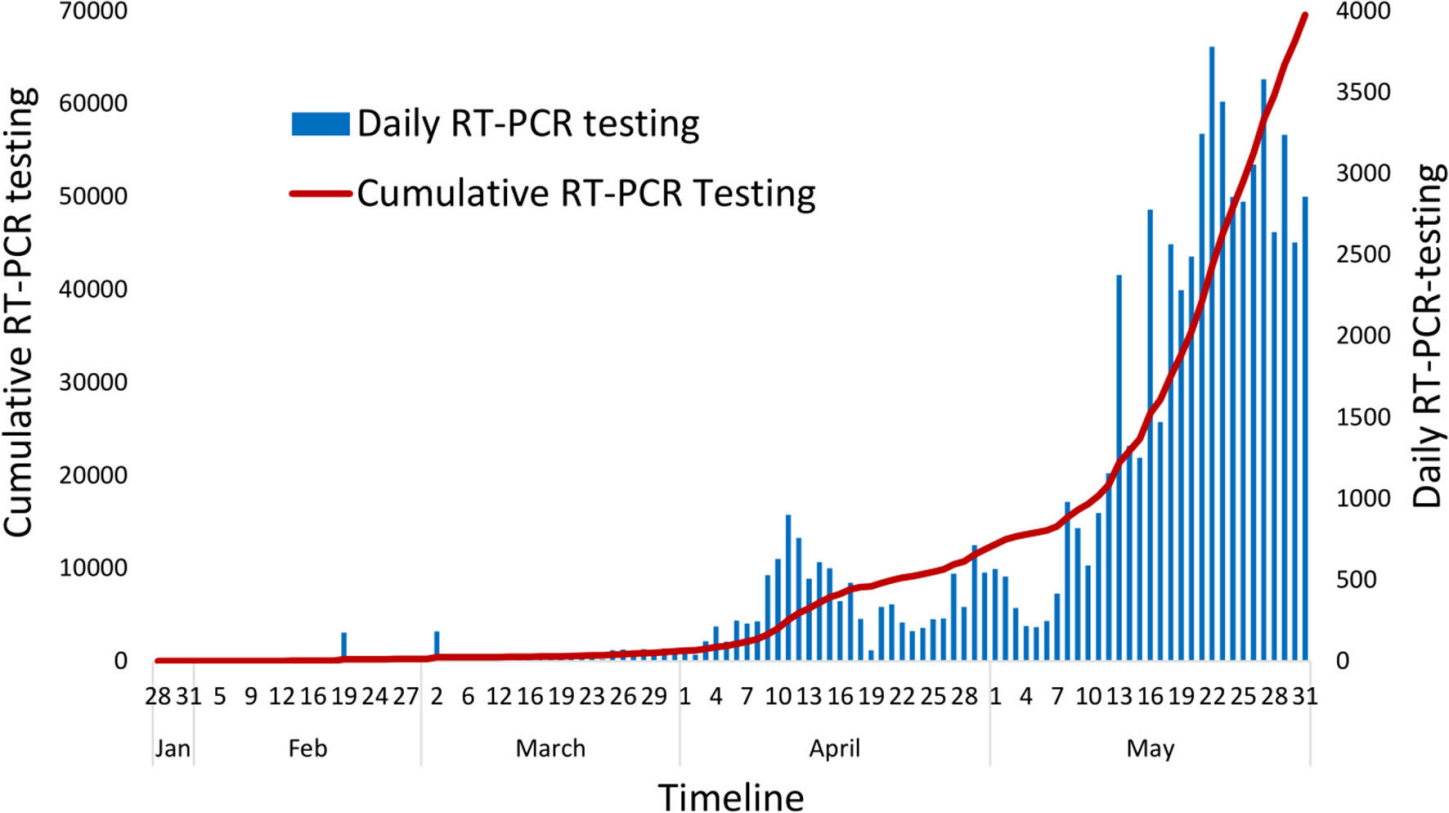
Cumulative and daily RT-PCR testing for COVID-19 diagnosis in Nepal.

The number of COVID-19 confirmed cases in Nepal reached 1,572 by May 31, 2020, after it was first confirmed in the country on January 23, 2020. The first case included a student who had returned from Wuhan, China, who, being aware of the coronavirus outbreak, visited the hospital in Kathmandu for a medical check-up (28). Among the total infected, 220 individuals were discharged from the hospital after testing negative. The epidemic curve based on daily data showed that cases have started rapidly rising since May 2020 (Figure 1). During this same period, 69,587 samples were tested using RT-PCR in 20 laboratories distributed across the country, with the majority of the tests being conducted at the National Public Health Laboratory (NPHL) based in the capital city, Kathmandu (Figure 2). This early epidemiological data indicates that the prevalence of COVID-19 among the tested individuals in Nepal was 2.25% (95% CI: 2.15–2.37%) (*n* = 1,572/69,587). More than 95% of these cases were asymptomatic. The tested individuals were mostly people who came in contact with the confirmed cases identified through contact tracing or those in quarantine set up by the government who had returned from foreign countries, the majority of whom returned from India. The earlier cases in Nepal up to mid-April 2020 had a travel history from countries such as China, France, Qatar, Belgium, the United Arab Emirates, the United Kingdom, and Saudi Arabia. All cases after mid-April were either linked to people coming from India via land or people contracting the virus locally, as all international flights were closed effective from March 23, 2020.

### Age and Gender Distribution

Among the 1,572 confirmed cases, eight people (0.5%) had died from COVID-19 in Nepal by May 31, 2020. The first COVID-19 death was reported on May 16, 2020, in a new mother who gave birth to her child on May 6th, 2020 in a hospital in Kathmandu and was discharged. She went to her home in Sidhupalchok district, around 4-h bus travel from Kathmandu, and later developed signs of fever and respiratory difficulties and ultimately died on May 16, 2020. The majority of the other deaths were in quarantine and confirmed as COVID-19 after their deaths.

**Figure 3:**
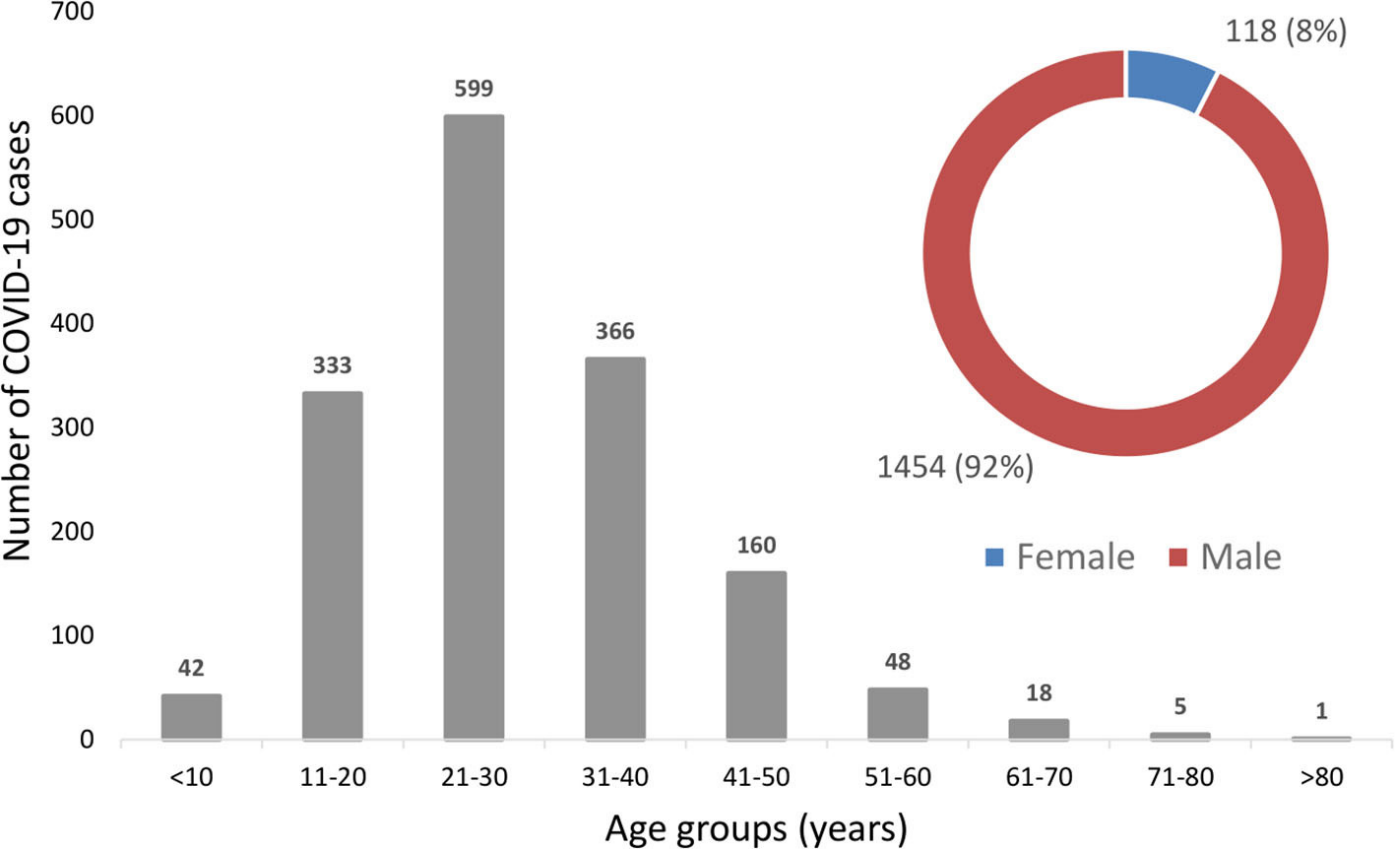
Age and gender-wise distribution of COVID-19 cases in Nepal.

The majority of the cases confirmed in Nepal were young males (Figure 3). 92% (*n* = 1,454/1,572) of the total cases were males and only 8% (*n* = 118/1,572) were females (Figure 3). This is not surprising as this population was tested most given their higher proportion in quarantine. The average age among the overall cases was 30.5 years (Range: 2 months to 81 years). Disaggregation by gender showed that the average age among the males was 30.4 years (Range: 2 months to 74 years) while the average age among the females was 30.8 years (Range: 4 months to 81 years), showing no statistical difference between the average ages by gender (*p* = 0.82). The detailed descriptive statistics of overall age distribution and gender are shown in Table 1.

**Table 1:** Age distribution of COVID-19 cases in Nepal^*^.

**COVID-19**	**Mean**	**Std. Dev**.	**Median**	**1st quartile**	**3rd quartile**
**cases**	**(Years)**	**(Years)**	**(Years)**	**(25%)**	**(75%)**
All (*N* = 717)	30.5	13.4	29	21	37
Male (*N* = 616)	30.4	12.3	29	22	36.5
Female (*N* = 101)	30.8	18.7	28	18	43

* *Out of 1,572 cases by May 31, 2020, exact age of 717 cases were made public while age of remaining cases were provided in ranges*.

### Spatial Patterns

**Figure 4:**
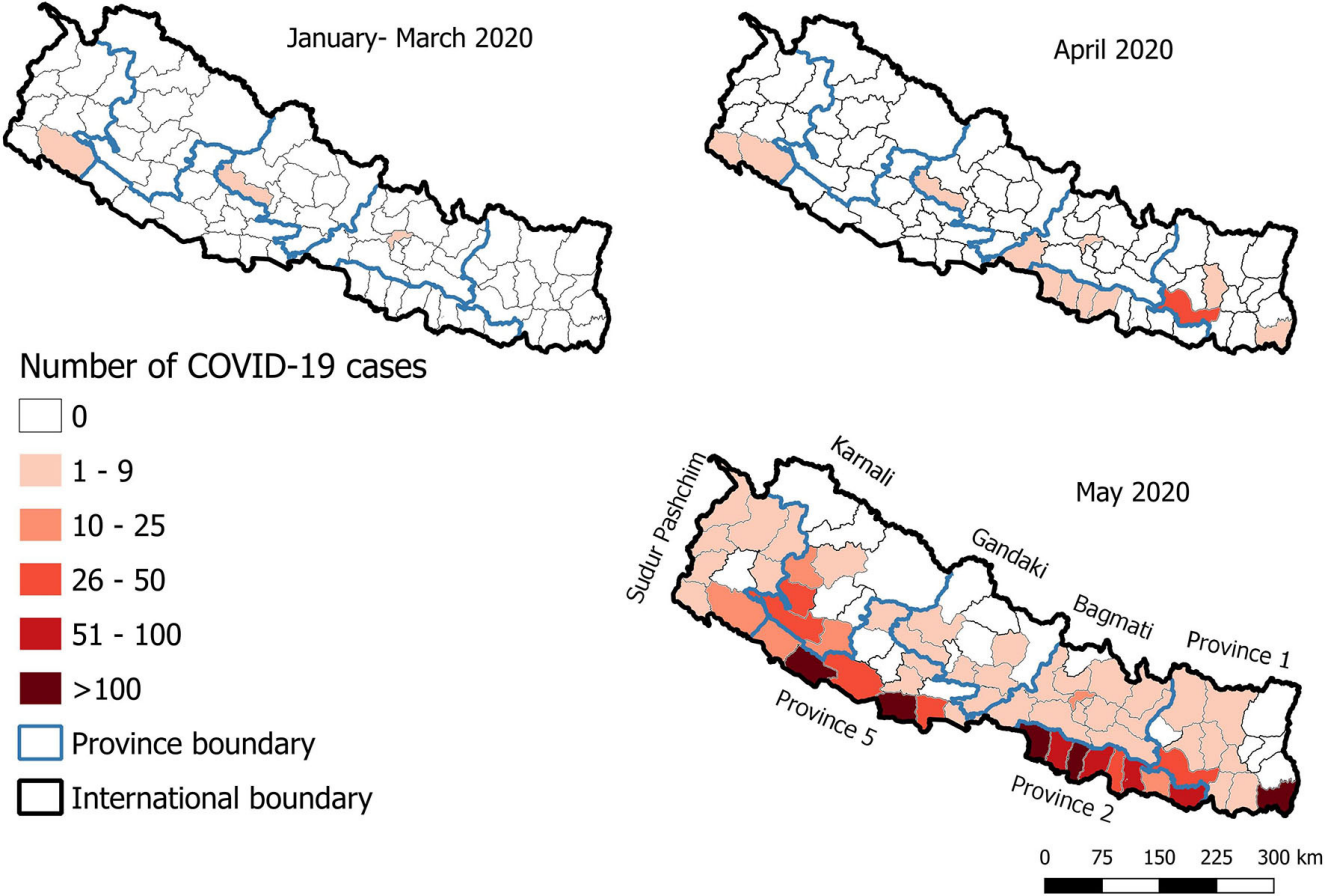
Spatial distribution of COVD-19 cases in Nepal.

The spatial pattern of COVID-19 cases in Nepal showed that, up to the end of March 2020, cases were reported only from three districts—Kathmandu, Baglung, and Kailali—out of 77 districts of Nepal. The number of districts affected increased to 12 by the end of April 2020 (Figure 4). Most of these districts had sporadic cases, except for Udayapur district in the eastern part of Nepal, where a cluster of cases (*n* = 28) was reported from one small village (Figure 4). The number of districts affected substantially increased and reached 57 out of 77 districts by the end of May 2020 (Figure 4). The majority of the cases were observed in the southern plains of Nepal bordering India in Provinces 1, 2, and 5. Five districts, namely Jhapa, Parsa, Rautahat, Banke, and Kapilvastu reported more than 100 confirmed cases (Figure 4). The province-wise distribution shows that Province 2 had the highest number of confirmed cases (*n* = 624 out of 1,572), followed by Province 5 (*n* = 565 out of 1,572), Province 1 (*n* = 165 out of 1,572), Karnali province (*n* = 123 out of 1,572), Bagmati province (*n* = 45 out of 1,572), Sudur Pashchim province (*n* = 27 out of 1,572), and Gandaki province (*n* = 23 out of 1,572).

### Public Health Measures Adopted by the Government

The Government of Nepal closed all international flights and its international borders on March 23, 2020, after the second COVID-19 case was recorded in Nepal. A day after this, Nepal enforced a nation-wide lockdown on March 24, 2020, which has been extended continuously and has been in effect up to June 14, 2020. The total length of continuous lockdown shall reach 83 days by June 14, 2020. The lockdown modality after June 14, 2020, is not clear at the moment of drafting this manuscript. The government has been using both RT-PCR and antibody-based rapid diagnostic tests (RDT) in parallel to diagnose or screen probable patients. However, only RT-PCR has been considered the confirmatory test. The government increased the number of RT-PCR testing laboratories from one to 20, including four veterinary laboratories. By May 31, 2020, the government had used 111,109 RDT tests to screen people in quarantine or other suspected areas. It has been a challenge for all three tiers of the Government, Federal, Provincial, and Local, to manage the large influx of Nepalese people wanting to return home from India. A small subset of people who have already entered Nepal has been kept in quarantine, which is often reported to be poorly managed due to limited resources. Up till now, the government has been isolating all COVID-19 cases in designated COVID-19 hospitals, irrespective of their clinical situation. This has overwhelmed hospitals with patients who do not need immediate medical attention.

## Discussion

This study was conducted to describe the spatiotemporal patterns and early epidemiological features of COVID-19 cases in Nepal from January 23 to May 31, 2020. The findings show that the vast majority of the cases in Nepal were young males and the case fatality rate was 0.5%. The disease was rapidly spreading and reached 57 out of 77 districts from all seven provinces by the end of May 2020.

The strict lockdown, meticulous testing and tracking, and massive isolation of people helped China to reduce the effects of the COVID-19 pandemic (29). Precise and widespread contact tracing and testing, including of asymptomatic individuals, together with social distancing led Taiwan to control COVID-19 in a fascinating way (30, 31). Similar intensive measures were also successfully used by South Korea to reduce COVID-19-associated casualties (32). Likewise, Vietnam, a country of 97 million people with limited resources, has been successful in limiting the spread of COVID-19 through a strong response system, including quick strategic testing and aggressive contact tracing (33). Nepal closed its international borders and enforced a country-wide lockdown early on, when only two cases were identified. The non-pharmaceutical interventions, including border control, lockdown, social distancing, and personal hygiene, helped Nepal in preventing the spread of SARS-CoV-2 during the initial days. However, later on, the effectiveness of the countrywide lockdown has not been observed, as the number of cases surged from 57 cases up to April to 1,572 by the end of May 2020 (25). One major contributor to this surge has been the return of daily wage migrant workers from India (34), where the cases of COVID-19 has been rapidly increasing since April 2020 (10). As Nepal shares its open border with India, citizens desperate to return home found different ways to return to Nepal, including swimming across the Mahakali river bordering two countries (35). There was also significant in-country movement of people wanting to return to their hometown as their livelihood sources in cities were compromised due to the lockdown.

Based on the available data, we estimated the COVID-19 prevalence in Nepal to be 2.25% (n =1,572/69,587). However, it may not represent the actual COVID-19 prevalence because samples from COVID-19 positive individuals are tested at least twice before declaring them COVID-19 negative and added in total numbers, without separating them. This prevalence also might not represent national level prevalence as samples from random populations have not been tested. Early studies reported that COVID-19 patients in Nepal showed few or no symptoms at all (24, 36). The situation updates of the Ministry of Health and Population (MoHP) also indicates that most of the confirmed cases are found through active surveillance and contact tracing rather than patients visiting hospitals with symptoms (25). This is in contrary to what is observed in other countries. The reported death rate (0.5%, n =8/1,572) also appears lower in comparison to the case-fatality rates reported from other countries. As per the mortality analysis carried out by Johns Hopkins University, among the 20 countries most severely affected with COVID-19 as of June 5, 2020, the case-fatality rate is highest in France (15.3%) and lowest in Chile (1.1%) (37). Nepal's neighboring countries, including China, India, Pakistan, and Bangladesh, have 5.5, 2.8, 2.1, and 1.4% case-fatality rates, respectively (37). Though the case fatality rates seem lower, it should not contribute to the relaxing of ongoing pandemic mitigation efforts by the Government of Nepal. The complete genome sequencing of the first SARS-CoV-2, isolated from Nepal, showed more than a 99% sequence homology with viruses isolated from Wuhan, China (38). Further studies are necessary to determine the origin and nature of SARS-CoV-2 circulating in Nepal. Importantly, the true burden of COVID-19 in South Asia, including Nepal, is difficult to estimate due to the low amount of testing and poor documentation (39). Moreover, as of May 31, 2020, the WHO classified the transmission pattern in Nepal as sporadic (18), which means Nepal has not yet observed the larger outbreaks of community-level transmission or the peak of the disease, which might be on its way. There are early signs of it as the WHO Nepal office has indicated that there is some evidence of secondary community transmission and a cluster of cases have been observed in four out of seven provinces of Nepal (40).

Nepal represents a real scenario of low- and middle- income countries (LMICs) where pandemic mitigation efforts are impacted largely by the lack of medical supplies and infrastructure. This includes personal protective equipment (PPE) and ventilators, the limitation of well-trained manpower, the unavailability of enough diagnostic kits; a lack of a proper coordination mechanisms among stakeholders, and poor reporting and documentation of cases (41–44). This pandemic has taught Nepal that it should invest more in research and development in the public health sector, besides the current primary focus on curative medicine. Current use of the laboratory facilities developed by the veterinary sector, to tackle with periodic disease outbreaks in animals including avian influenza viruses (45), for COVID-19 diagnostic purposes further highlights the necessity of intersectoral collaboration in pandemic mitigation efforts. A multisectoral and collaborative one-health approach including animal health, human health, and environmental health professionals (46) will not only be effective in managing the ongoing COVID-19 pandemic control but also will allow for better preparedness against future outbreaks and other imminent problems, such as antimicrobial resistance in Nepal.

COVID-19 has geographically expanded and affected all age groups in Nepal. As of June 6, 2020, the total number of cases and deaths have reached 3,235 and 13, respectively, from 69 out of 77 districts of Nepal (25). The Government of Nepal has been using lockdown as one of its major weapons against COVID-19. If enforced correctly, lockdown measures can effectively reduce the spread of the virus (47). However, the enforcement of a lockdown will likely be less effective if it is continued for long periods of time. Besides this tactic, the government should also consider and be prepared for managing the socio-cultural, economical, and psychological burdens of the lockdown, if it will be continued further. It will be very challenging for countries like Nepal to opt for indefinite lockdown measures given their limited resources and vulnerable socio-economic status.

### Strength and Limitations

The strength of this study is that it uses the daily data made public by the MoHP and provides early epidemiological features of COVID-19 cases in Nepal. This study will provide a baseline to compare the epidemiological features of COVID-19 cases in Nepal in the future, as the pattern might change with progression in infection. As only RT-PCR confirmed cases were included in the study, the data is reliable and provides useful information regarding the spatiotemporal patterns of COVID-19 cases in Nepal. However, this study has some limitations, such as the prevalence calculated in this study perhaps being an underestimation as the number of individuals tested is lower than the total samples tested. In addition, the estimated prevalence is only a reflection of those who are tested rather than the true prevalence at the population level.

## Conclusion and Recommendation

This study provides an overview of the spatiotemporal patterns and early epidemiological features of COVID-19 cases in Nepal. There were 1,572 cases and eight deaths associated with COVID-19 in Nepal by the end of May 2020. The estimate of prevalence for COVID-19 among the tested population was 2.25% and case-fatality rate was 0.5%. The majority of the cases were young and were mostly asymptomatic. The disease had spread to 57 out of 77 districts of Nepal by the end of May 2020, despite the continuous lockdown.

Moving forward, it would be better to identify high-, medium-, and low-risk areas and make appropriate plans for loosening lockdowns in a phase-wise manner to return toward the state of “new normal.” As the effect of COVID-19 is likely to persist longer (48), practice of social distancing and good personal hygiene, including the use of face masks, continuous scrutiny at the porous Indian border, increased testing, tracking, and medical capacity, and proper quarantine of cases and high-risk groups should continue in Nepal.

## Data Availability Statement

All datasets generated for this study are included in the article/supplementary material.

## Author Contributions

SD and SK conceived the idea and designed the study and prepared the first draft and revised it. SK collected and analyzed the data. All authors have approved the final version.

## Conflict of Interest

The authors declare that the research was conducted in the absence of any commercial or financial relationships that could be construed as a potential conflict of interest.
